# Glycemic Control and Hypoglycemia in Patients Treated with Insulin Pump Therapy: An Observational Study

**DOI:** 10.1155/2020/1581726

**Published:** 2020-08-05

**Authors:** Guillermo Guzmán, Veline Martínez, Julián David Yara, Miguel Angel Mina, Juan Sebastian Solarte, Angela María Victoria, Karen Fériz

**Affiliations:** ^1^Fundación Valle del Lili, Departamento de Endocrinología, Cra 98 No. 18-49, Cali 760032, Colombia; ^2^Universidad Icesi, Facultad de Ciencias de la Salud, Calle 18 No. 122-135, Cali, Colombia; ^3^Fundación Valle del Lili, Departamento de Medicina Interna, Cra 98 No. 18-49, Cali 760032, Colombia

## Abstract

**Introduction:**

Diabetes mellitus (DM) is a highly prevalent disease worldwide. It has been associated with an important morbimortality due to its complications and sometimes as a result of adverse events related to treatment. Insulin pump therapy (IPT) is one of the options used to control this disease and reduces one of the most frequent complication associated with treatment: hypoglycemia, which has also a great impact on life quality and clinical status of patients.

**Materials and Methods:**

A descriptive and retrospective study was performed including patients treated and followed by the department of endocrinology from a high-complexity university hospital in Cali, Colombia, between 2012 and 2017. Patients were on IPT and continuous glucose monitoring (CGM): MiniMed Paradigm® Veo™ Insulin Pump (Medtronic®) and MiniMed 640G Insulin Pump-Enlite™ Sensor (Medtronic®). Presentation of hypoglycemia and variables associated with its development were evaluated.

**Results:**

51 patients were included. The main indication for IPT initiation was the report of hypoglycemic episodes and inappropriate metabolic control. Initiation of IPT was related with a decrease in glycosylated hemoglobin (HbA1c) and also a decrease in severe hypoglycemic events and hospitalization due to hypoglycemia. The risk factors linked with clinically significant hypoglycemia were male gender, and standard deviation of glucose measures calculated by CGM. A diminished glomerular filtration rate (GFR) (<60 mL/min/1.73 m^2^) was correlated with higher risk of severe hypoglycemia.

**Conclusion:**

IPT with CGM is a useful strategy in the management of patients with DM; it is associated with a reduction of adverse hypoglycemic events and hospitalizations due to hypoglycemia.

## 1. Introduction

Diabetes mellitus (DM) has become a public health concern worldwide, its prevalence is on the rise, and it has been projected to have an increase of 10% by the year 2045 [1]. Currently in Colombia, 7.4% of men and 8.7% of women are diagnosed with type 2 diabetes mellitus (T2DM); meanwhile, the prevalence of type 1 diabetes (T1D) is lower and has been estimated around 0.07% [2]. Both T1D and T2DM have been associated with a significant number of micro- and macrovascular complications, resulting from inappropriate glycemic control, causing an important impact on life quality of patients and producing high costs for healthcare systems. Globally, up to 30% of those with DM have poor glycemic control [3, 4]. Local data from our health center reveals that only 53.7% of patients with DM have an appropriate glycemic control [5].

Hypoglycemia is a frequent complication derived from standard management of diabetes, enacting not only a barrier to achieve treatment goals but also a source of morbidity and mortality in patients with DM. The global Hypoglycemia Assessment Tool (HAT) study showed that up to 97.4% of type 1 diabetics and 95% of type 2 diabetics have at least one episode of hypoglycemia in a period of 4 weeks [6].

Given the difficulty to achieve certain treatment goals and due to the presence of hypoglycemia as a limiting factor, several strategies have been developed. As an example, there are insulin analogues which allow a better simulation of normal pancreatic insulin secretion and reduce in this way the rate of hypoglycemic events; however, in some patients, the goal of reducing glycosylated hemoglobin (HbA1c) without presenting hypoglycemic episodes continues to be unachieved. In those patients, insulin pump therapy (IPT) is a good choice since it has been shown that a better glycemic control is obtained with its usage and there is a significant reduction in hypoglycemic events [7–14].

Current evidence related to IPT has shown a reduction of the number of severe episodes of hypoglycemia, although the difference with nonsevere events remains a source of debate [15]. Likewise, this kind of therapy coupled with the use of continuous glucose monitoring (CGM) system has been associated with a reduction in moderate and severe hypoglycemic events [16]. Despite this, the frequency of events is still considerable as well as the complications derived from it. In this study, we aim to evaluate the factors that could be related to hypoglycemic events.

## 2. Materials and Methods

We performed an observational retrospective study at University Hospital Fundación Valle del Lili (FVL) in Cali, Colombia, from 2012 to 2017. FVL is a fourth-level healthcare center that serves as a reference facility for diabetic patients from the southwestern region of the country.

We included patients ≥18 years of age with diagnosis of DM who were followed-up at FVL endocrinology outpatient clinic. These individuals initiated their treatment in our institution or were referred to it due to administrative issues with health insurances. We excluded patients with incomplete variables or outcome data.

Eligible patients were users of subcutaneous IPT integrated to CGM: MiniMed Paradigm® Veo™ Insulin Pump (Medtronic®) and MiniMed 640G Insulin Pump-Enlite™ Sensor (Medtronic®). These patients received at least 6 months of treatment, had a measurement of HbA1c, and in their last control had downloaded data for the evaluation of hypoglycemic events of the last 14 days.

We reviewed data of 51 patients who met the eligibility criteria; demographic variables such as age, gender, educational level, and past medical history related to their condition such as type of diabetes and date of diagnosis were recorded. Furthermore, other relevant clinical variables were included such as weight, height, body mass index (BMI), indications for therapy initiation, and laboratory results.

Frequency and type of hypoglycemia were determined from the records of biweekly continuous glucose sensor downloads. Complications such as infection of catheter insertion site and episodes of diabetic ketoacidosis after system implantation were obtained from the medical registries.

For the evaluation of hypoglycemia, interstitial glucose data measured by continuous glucose sensor was used. Hypoglycemia was defined as levels below the threshold for a time greater than 15 minutes, considering a second event when it was detached from the first one for 120 minutes. A risk of hypoglycemia was defined as interstitial glucose less than 70 mg/dL but greater than 54 mg/dL; clinically significant hypoglycemia was considered when it was less than 54 mg/dL, and severe hypoglycemia for any glucose value with severe neurological compromise requiring third-party assistance to resolve the event [17].

### 2.1. Statistical Analysis

Data collected was analyzed with Stata 13 (Stata Corporation, College Station, TX, USA). The quantitative variables were reported as means and standard deviations or medians and interquartile ranges. The categorical variables were described as frequencies and percentages. For comparison, the Student *t*-test or Wilcoxon rank-sum test were used for continuous variables and *χ*2 test or Fisher's exact test were used for categorical variables according to the fulfillment of assumptions. A multivariate logistic regression model was used to evaluate associated factors with the presence of clinically significant hypoglycemia and severe hypoglycemia. This study was approved by the FVL institutional review board.

## 3. Results

**Figure 1:**
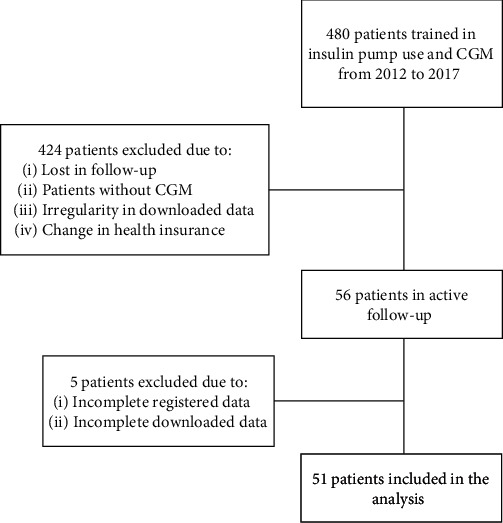
Flow diagram of patients in the study. CGM: continuous glucose monitoring.

From January 2012 to December 2017, 480 patients received guidance on the usage of IPT in FVL; from them, only 56 continued periodic monitoring in the institution of which 51 were definitely included in the analysis due to their complete data in medical registries (Figure 1). 64% were women, the average age was 40.98 ± 13.7 years, and 90.2% had type 1 diabetes (Table 1).

Regarding indications for IPT initiation, hypoglycemia was the most frequent one (21.5%), followed by high glycemic variability and poor glycemic control. The average time on IPT was 2 years, and the median time with diagnosis of DM was 16 years. The most frequent complications after the beginning of IPT were contact dermatitis and infection of the catheter insertion site which occurred on 23% and 11.7%, respectively (Table 1).

**Figure 2:**
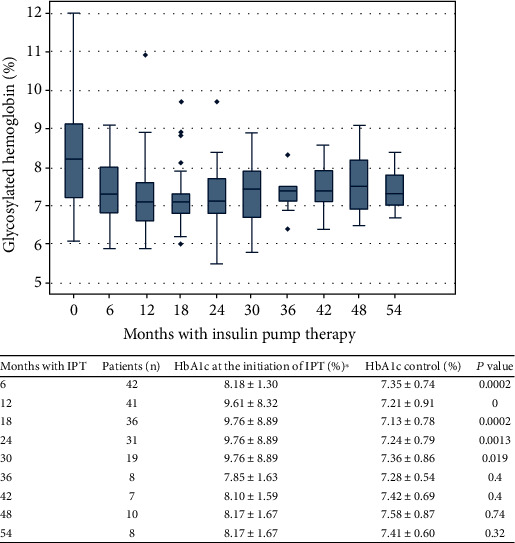
HbA1c levels prior to the beginning of insulin pump therapy (time 0) and during the next months (6 to 54 months). Results are reported as median values and interquartile ranges. In the table, we report the HbA1c level at the beginning of the therapy and its control after the initiation of IPT.

At the beginning of the therapy, patients had a median HbA1c of 8.21% (7.2-9.13), and at the sixth month of therapy, there was a decrease (0.7%, *P* = 0.0002) that was sustained over time (Figure 2). Patients presented high adherence to treatment, defined as percentage of continuous glucose sensor use greater than 80.3%. The use of Bolus Wizard (special feature from insulin pump to calculate food and bolus correction amount) was found in 96.6%. Patient's characteristics after the initiation of IPT are specified in Table 2. 88.24% of the patients presented at least one episode of risk of hypoglycemia and 62.75% one of clinically significant hypoglycemia. Severe hypoglycemia occurred in 29.16% of the patients, and 13.72% of them required hospitalization. The mean coefficient of variation and standard deviation of glucose measures calculated by CGM was 35.86 ± 9.2 and 62.21 ± 20.02, respectively. After the onset of IPT, there was a significant reduction in hospitalizations due to hypoglycemia (*P* = 0.014) and severe hypoglycemia episodes (*P* = 0.0396) (Table 3).

The multivariate logistic regression model showed that male gender, daily insulin dosing, duration of the therapy, coefficient of variation, and standard deviation of glucose measures calculated by CGM were the most related factors to the presence of clinically significant hypoglycemia (Table 4). Men had 13-fold increased risk of this type of event compared to women (*P* = 0.013), and the standard deviation of glucose measures calculated by CGM was documented as a risk factor for clinically significant hypoglycemia (OR 1.11, 95% CI 1.0122-1.221, *P* = 0.027).

**Figure 3:**
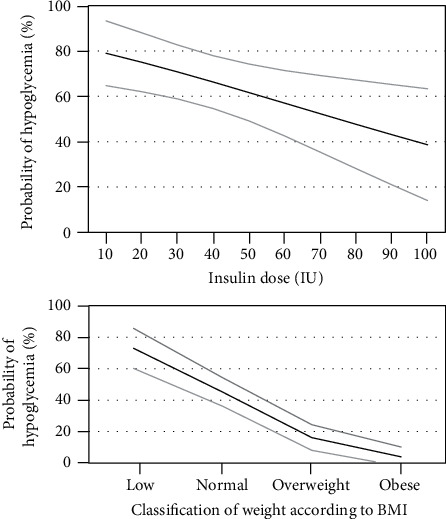
Probability of clinically significant hypoglycemia according to insulin dose and probability of severe hypoglycemia according to body mass index (BMI). The gray lines represent the 95% confidence interval.

Low coefficient of variation behaved as a protective factor, showing that the lower it was, the lower the risk of hypoglycemia. There was also an inverse relationship between daily insulin dosing and the development of this type of events. Regarding the presence of severe hypoglycemia, it was found that BMI at the onset of therapy was a protective variable, showing that patients with lower weight presented a greater number of hypoglycemic events. Decreased glomerular filtration rate (GFR) prior to the beginning of IPT was a risk factor for this type of event (Figure 3, Table 4).

## 4. Discussion

IPT and CGM have become an important tool for diabetes treatment, reducing HBA1c, hypoglycemic events, and associated complications [18, 19]. Despite it being a costly therapy, its availability has been increasing for the general population, an important aspect in our healthcare center due to 49% of the patients followed-up in the outpatient clinic belong to the lowest socioeconomic stratums.

Bergenstal et al. presented in their study a reduction of 0.2-0.4% in HbA1C with this therapy, and when IPT was coupled to CGM, this reduction could reach 0.6% with the advantage of allowing the patient to know the real-time glucose levels and make early decisions, for example, in daily insulin dosing [11, 16]. In our study, the reduction of HbA1c was 0.7% at the sixth month of treatment compared to the one taken before the onset, slightly lower than the one reported by Gómez et al. in Colombia and similar to the changes reported by other groups worldwide [17]. It is interesting how low levels of HbA1c persist in the long-term follow-up which could be influenced by the high frequency of continuous glucose sensor usage (>80%) and Bolus Wizard feature (>96%), found in our study. It has been demonstrated that the use of these tools is directly related to a reduction of HbA1c and hypoglycemic events when compared to the non-use of them [18–20].

In our cohort of patients, the main indication for beginning IPT was hypoglycemia. After the initiation of the treatment, a significant reduction of severe hypoglycemic events was achieved (*P* = 0.0396) in addition to a decrease in hospitalizations due to hypoglycemia (*P* = 0.014). Furthermore, it is known that IPT associated to its shut-off system decreases global hypoglycemia by 35% (*P* < 0.0001) and nocturnal hypoglycemia by 40% (*P* < 0.001); however, data regarding risk of hypoglycemia and nocturnal hypoglycemia was not available prior to the onset of therapy, which did not allow us to compare changes related to these variables when we implemented the treatment [21]. Nocturnal hypoglycemia was found in 72.55% of the patients, a slightly lower proportion than that reported in other studies [22].

Frequency of risk of hypoglycemia (88.24%) and clinically significant hypoglycemia (62.75%) was higher than the reported in other studies such as the ASPIRE In-Home study; nevertheless, the population is not comparable due to the one analyzed in the ASPIRE In-Home study differs from ours. Participants included minors, only T1 diabetic patients, and individuals without past medical history of hypertension or chronic kidney disease; additionally, they used a different definition for hypoglycemic event [23].

Determining the factors associated with clinically significant hypoglycemia, it was found that men presented 13-fold increased risk compared to women. Our results may be influenced by the lower number of men in the analyzed sample; nonetheless, they are comparable to the results found by the Diabetes Control and Complications Trial where the relative risk of hypoglycemia in intensive therapy was higher in men compared to women (4.35, 95% CI 3.20-5.90 vs. 2.52, 95% CI 1.87-3.38) [24].

We found that daily insulin dose has a slightly protective effect on the development of significant hypoglycemia, which could be related to the duration of therapy, use of the continuous glucose sensor, and the patient's care measures with higher doses of insulin. Another possible reason to this relation is that patients who require a higher amount of insulin could have an underlying condition causing insulin resistance. A prediction model established that the risk of hypoglycemia is directly proportional to increases in the total insulin dose until reaching a threshold of 0.8 units/kg where this relationship can no longer be observed [25–27]. Studies with a greater sample that show a stronger association are necessary.

Coefficient of variation and standard deviation of glucose measures calculated by CGM have been described as ways of representing glycemic fluctuation. Diverse studies have demonstrated the association between the coefficient of variation and adequate control of HbA1C or presence of hypoglycemia [27–29]. By prediction models, it has been established that for every 10% increase in the value of coefficient of variation, risk of hypoglycemia increases by 19% [27]. In our study, it was found that a low coefficient constitutes a protective factor to develop hypoglycemia. Also, the standard deviation of glucose measures calculated by CGM was significantly associated with the development of clinically significant hypoglycemia (OR 1.11 95% CI 1.0122-1.221); however, these two coefficients showed no significant relationship with severe hypoglycemia which could be limited by the sample of the study.

On the other hand, initial BMI had a protective effect for these events; the higher the BMI, the lower the probability of developing severe hypoglycemia, although the number of patients with low weight was very scarce which may have influenced the results.

Renal function also plays an important role in being at risk of hypoglycemia. In our study, we found that patients who had a decreased GFR at the onset of therapy had a higher risk of severe hypoglycemia. In a retrospective study conducted by Moen et al., it was demonstrated that GFR < 60 mL/min/1.73 m^2^ constitutes a risk factor for developing hypoglycemia; there is a 3-, 7-, and 8-fold risk for hypoglycemia with a GFR of 70, 60, and 50 mg/dL, respectively [30]. This could be explained by a decrease in renal gluconeogenesis and a diminished release of epinephrine as a counterregulatory hormone due to autonomic neuropathy in renal failure.

More studies are required to seek deeper for factors involved with the presence of hypoglycemia in patients with IPT. Limitations of this study include its sample size and difference in treatment times between patients due to lost in follow-up of individuals and administrative reasons beyond our institution, despite being a specialized center in the training and management of patients with this type of therapy. Likewise, there were no data regarding risk of hypoglycemia, nocturnal hypoglycemia, and clinically significant hypoglycemia prior to the therapy, since many patients started their follow-up in our institution after they had already initiated this type of treatment.

## 5. Conclusions

IPT associated with a CGM system is an effective strategy in the management of patients with DM, allowing better glycemic control with significant reduction of HbA1c levels and significant reduction in the risk of hypoglycemia. Several variables associated with the presence of hypoglycemia as well as protective factors were identified, which can constitute intervention targets in order to reduce the number of events and their clinical implications.

## Figures and Tables

**Table 1:** Sociodemographic and clinical characteristics of the patients prior to the beginning of insulin pump therapy.

Feature	Measure
Age, SD^∗^	40.98 ± 13.7
Gender, *n* (%)	
Female	33 (64.71)
Male	18 (35.29)
Socioeconomic stratum^∗∗∗^, *n* (%)	
1–2 (low)	25 (49)
3–4 (middle)	17 (33.3)
5–6 (high)	9 (17.6)
Educational level, *n* (%)	
Elementary school	1 (1.96)
High school	10 (19.61)
Technician/technologist	12 (23.53)
Professional	18 (35.29)
Specialization/master´s	10 (19.61)
Type of diabetes, *n* (%)	
Type 1	46 (90.2)
Type 2	3 (5.9)
Other	2 (3.9)
Time since diagnosis of diabetes (months)^∗∗^	197 (100-336)
Weight (kg)^∗∗^	63.2 (56.9-69.5)
Height (cm)^∗∗^	161.5 (148-166.5)
BMI^∗∗^	24 (22.39-26.1)
Total daily insulin dose (IU)^∗∗^	39 (30-60)
HbA1c (%)^∗∗^	8.21 (7.2-9.13)
Indication to start insulin pump therapy, *n* (%)	
Hypoglycemia	11 (21.6)
High variability	9 (17.6)
Poor metabolic control	7 (13.7)
Hypoglycemia and high variability	9 (17.6)
Hypoglycemia and poor metabolic control	9 (17.6)
Poor metabolic control and high variability	3 (5.9)
Hypoglycemia, poor metabolic control and high variability	3 (5.9)
Type of insulin pump	
MiniMed Paradigm® Veo™ (Medtronic®)	43 (84.3)
MiniMed 640G - Enlite™ Sensor (Medtronic®)	8 (15.7)

^∗^Reported as mean value ± SD: standard deviation. ^∗∗^Reported as median value (interquartile range); *N* (%): number (percentage); BMI: body mass index; IU: international units; HbA1c: glycosylated hemoglobin. ^∗∗∗^Socioeconomic stratum based on the socioeconomic distribution of households per neighborhood in Colombia.

**Table 2:** Characteristics of patients after the initiation of insulin pump therapy.

Variable	Measure
Time with insulin pump therapy (months)^∗∗∗^	24 (18-49)
Weight (kg)^∗∗∗^	64 (56-72)
BMI^∗∗∗^	24.8 (22.25-27.5)
HbA1c posterior to the initiation of IPT (%)^∗∗∗^	7.5 (6.9–8.1)
Insulin dose (IU)^∗∗∗^	37 (27.4-51.1)
Use of continuous glucose sensor (%)	80.3
Distribution of insulin use^∗∗∗^	
Basal	51 (43-57)
Boluses	49 (43-57)
Units of basal insulin	4 (3-5)
Units of correction insulin	90 (67.4-96.6)
Overcorrection^∗∗^	
Yes	7 (13.7)
Not	44 (86.2)
Coefficient of variability (%)^∗^	35.86 ± 9.2
SD of glucose measures calculated by CGM (mg/dL)^∗^	62.21 ± 20.02
Complications associated to the use of IPT^∗∗^	
None	28 (55.5)
Irritation at the catheter insertion site	12 (23)
Infection at the catheter insertion site	6 (11.7)
Diabetic ketoacidosis	5 (9.8)

^∗^Reported as mean value (standard deviation). ^∗∗^Values reported as absolute number, *n* (percentage). ^∗∗∗^Reported as median (interquartile range). BMI: body mass index; HbA1c: glycosylated hemoglobin; IPT: insulin pump therapy; IU: international units; SD: standard deviation.

**Table 3:** Events related to hypoglycemia prior and after the initiation of insulin pump therapy.

Events	Prior to the initiation of IPT	After the initiation of IPT	*P* value
Risk of hypoglycemia, *n* (%)	∗		—
0		6 (11.76)	
1-5		33 (64.7)	
>5		12 (23.54)	
Clinically significant hypoglycemia, *n* (%)	∗		—
0		19 (37.25)	
1-5		27 (52.94)	
>5		5 (9.81)	
Hospitalization due to hypoglycemia, *n* (%)			0.014^∗^
Yes	16 (28.57)	7 (13.72)	
No	35 (71.43)	44 (86.27)	
Severe hypoglycemia, *n* (%)			0.0396^∗^
Yes	28 (54.9)	17 (29.16)	
No	23 (45.1)	34 (70.83)	
Nocturnal hypoglycemia, *n* (%)	∗		—
Yes		37 (72.55)	
No		14 (27.45)	
Fear of hypoglycemia related to, *n* (%):	∗	24 (47.06)	
Risk of hypoglycemia			0.318
Clinically significant hypoglycemia			0.743
Severe hypoglycemia			0.502
Hospitalization due to hypoglycemia			1
Nocturnal hypoglycemia			0.472
Perception of hypoglycemia related to, *n* (%):	∗	38 (74.51)	
Risk of hypoglycemia			1
Clinically significant hypoglycemia			0.691
Severe hypoglycemia			0.692
Hospitalization due to hypoglycemia			0.703
Nocturnal hypoglycemia			0.541
Count of CH, *n* (%):	∗		—
CH counting tables		8 (15.69)	
CH weighing		3 (5.88)	
Approximation/experience		40 (78.43)	
Count and relation to			
Risk of hypoglycemia			0.352
CH counting tables		7 (15.6)	
CH weighing		2 (4.4)	
Approximation/experience		36 (80)	
Clinically significant hypoglycemia			0.385
CH counting tables		5 (15.6)	
CH weighing		3 (9.4)	
Approximation/experience		24(75)	
Severe hypoglycemia			1
CH counting tables		3 (17.6)	
CH weighing		1 (5.9)	
Approximation/experience		13 (76.5)	
Hospitalization due to hypoglycemia			0.179
CH counting tables		2 (28.6)	
CH weighing		1 (14.3)	
Approximation/experience		4 (57.1)	
Nocturnal hypoglycemia			0.515
CH counting tables		5 (13.5)	
CH weighing		3 (8.1)	
Approximation/experience		29 (78.4)	

∗No available data. IPT: insulin pump therapy; CH: carbohydrates. ^∗^*P* < 0.05.

**Table 4:** Variables related to hypoglycemia.

Variables	OR	95% CI	*P* value
Clinically significant hypoglycemia			
Male gender	13.03	1.700-9.831	0.013
Daily insulin dose	0.97	0.947–0.997	0.048
Time receiving IPT	1.03	0.996-1.070	0.081
Coefficient of variation	0.75	0.587-0.9742	0.031
SD of glucose measures calculated by CGM	1.11	1.0122-1.221	0.027
Severe hypoglycemia			
Initial BMI	0.02	0.001-0.348	0.006
AUC > 140	1.09	1.014-1.188	0.021
Number of basal boluses of insulin	0.49	0.244-1.008	0.053
Initial GFR < 60	58.85	3.287-10.535	0.006
SD of glucose measures calculated by CGM	1.00	0.808-1.239	0.995

OR: odds ratio; CI: confidence interval; IPT: insulin pump therapy; SD: standard deviation; CGM: continuous glucose monitoring; BMI: body mass index; AUC: area under the curve of glucose; GFR: glomerular filtration rate (expressed in mL/min/1.73 m^2^).

## Data Availability

The data used to support the findings of this study are restricted by Fundación Valle del Lili Ethics Committee in order to protect patient privacy. Data are available from Dr. Guillermo E. Guzmán (contact: guillermoeguzman@gmail.com), for researchers who meet the criteria for access to confidential data.
